# MicroRNA-223-3p promotes skeletal muscle regeneration by regulating inflammation in mice

**DOI:** 10.1074/jbc.RA119.012263

**Published:** 2020-06-03

**Authors:** Naixuan Cheng, Chang Liu, Yulin Li, Shijuan Gao, Ying-Chun Han, Xiaonan Wang, Jie Du, Congcong Zhang

**Affiliations:** 1 School of Basic Medical Sciences, Capital Medical University, Beijing, China; 2 Beijing Anzhen Hospital, Affiliated to Capital Medical University, Beijing Institute of Heart, Lung and Blood Vessel Diseases, Beijing, China; 3 Renal Division, Department of Medicine, Emory University, Atlanta, Georgia, USA

**Keywords:** muscle regeneration, inflammation, miR-223-3p, macrophage, cytokines, epigenetics, muscle satellite stem cell, small noncoding RNA, cytokine, interleukin-6 (IL-6)

## Abstract

After injury, the coordinated balance of pro- and anti-inflammatory factors in the microenvironment contribute to skeletal muscle regeneration. However, the underlying molecular mechanisms regulating this balance remain incompletely understood. In this study, we examined the roles of microRNAs (miRNAs) in inflammation and muscle regeneration. miRNA-Seq transcriptome analysis of mouse skeletal muscle revealed that miR-223-3p is upregulated in the early stage of muscle regeneration after injury. miR-223-3p knockout resulted in increased inflammation, impaired muscle regeneration, and increased interstitial fibrosis. Mechanistically, we found that myeloid-derived miR-223-3p suppresses the target gene interleukin-6 (*Il6*), associated with the maintenance of the proinflammatory macrophage phenotype during injury. Administration of IL-6-neutralizing antibody in miR-223-3p-knockout muscle could rescue the impaired regeneration ability and reduce the fibrosis. Together, our results reveal that miR-223-3p improves muscle regeneration by regulating inflammation, indicating that miRNAs can participate in skeletal muscle regeneration by controlling the balance of pro- and anti-inflammatory factors in the skeletal muscle microenvironment.

In skeletal muscle, the capacity for regeneration and repair after injury depends on muscle satellite stem cells (MuSCs), a population of myogenic precursor cells found on the surface of the epicardium and perimysium. After their activation by injury, MuSCs enter the cell cycle and proliferate to produce a large number of cells. Some of these return to a quiescent state to maintain the reserve pool, whereas others continue to differentiate, fusing with residual muscle fibers to form new myofiber. During skeletal muscle regeneration, MuSCs are regulated by cues from the niche microenvironment, especially inflammatory cells and the cytokines they secrete (1–3).

Macrophages play an indispensable role in skeletal muscle regeneration. During the inflammatory phase after skeletal muscle injury, Ly6C^hi^ proinflammatory macrophages accumulate and act as phagocytes to clear necrotic myofibers and promote myoblast proliferation. Subsequently, these proinflammatory macrophages transformed into a Ly6C^lo^ phenotype and secrete anti-inflammatory cytokines to terminate the local inflammatory response and promote myoblast differentiation and fusion and the formation of new muscle fibers (2, 4–7). The transformation of macrophages from the proinflammatory phenotype to the anti-inflammatory phenotype changes the expression profiles of pro- and anti-inflammatory cytokines, further influencing the inflammatory response. For example, interleukin-6 (IL-6), a cytokine highly expressed by proinflammatory macrophages, stimulates expression of the chemokines C-C motif chemokine ligand 2 (CCL2) and CCL3, enabling the recruitment of more inflammatory macrophages from the circulation and facilitating muscle regeneration (8). However, continuously high expression of IL-6 results in the accumulation of proinflammatory macrophages in muscle and high levels of the catabolic signals serum amyloid A and fibrinogen, resulting in muscle atrophy (9, 10). Thus, timely and orderly transformation of the pro-/anti-inflammatory microenvironmental balance is important for effective skeletal muscle regeneration after injury. However, the mechanism regulating this balance remains to be elucidated.

MicroRNAs (miRNAs) are evolutionarily conserved, small noncoding RNAs of 18–22 nucleotides in length. They have been reported to regulate skeletal muscle regeneration (11, 12), and some miRNAs (miR-155 and miR-21) could regulate macrophage differentiation during this process (13, 14). However, the understanding of the roles of miRNAs in skeletal muscle regeneration remains incomplete.

miRNA transcriptome analysis of mouse skeletal muscle revealed that miR-223-3p is upregulated significantly in the early stage of muscle regeneration after injury, but the role of miR-223-3p in muscle regeneration after injury is still unknown. In this study, we show that miR-223-3p knockout mice display impaired muscle regeneration and increased interstitial fibrosis, as well as increased inflammation. Mechanistically, we found that miR-223-3p was linked to its target gene, *Il6*, which activates macrophage differentiation into proinflammatory subtypes during the early inflammatory response after muscle injury.

## Results

### miR-223-3p is upregulated in skeletal muscle in early stages after injury

**Figure 1.**
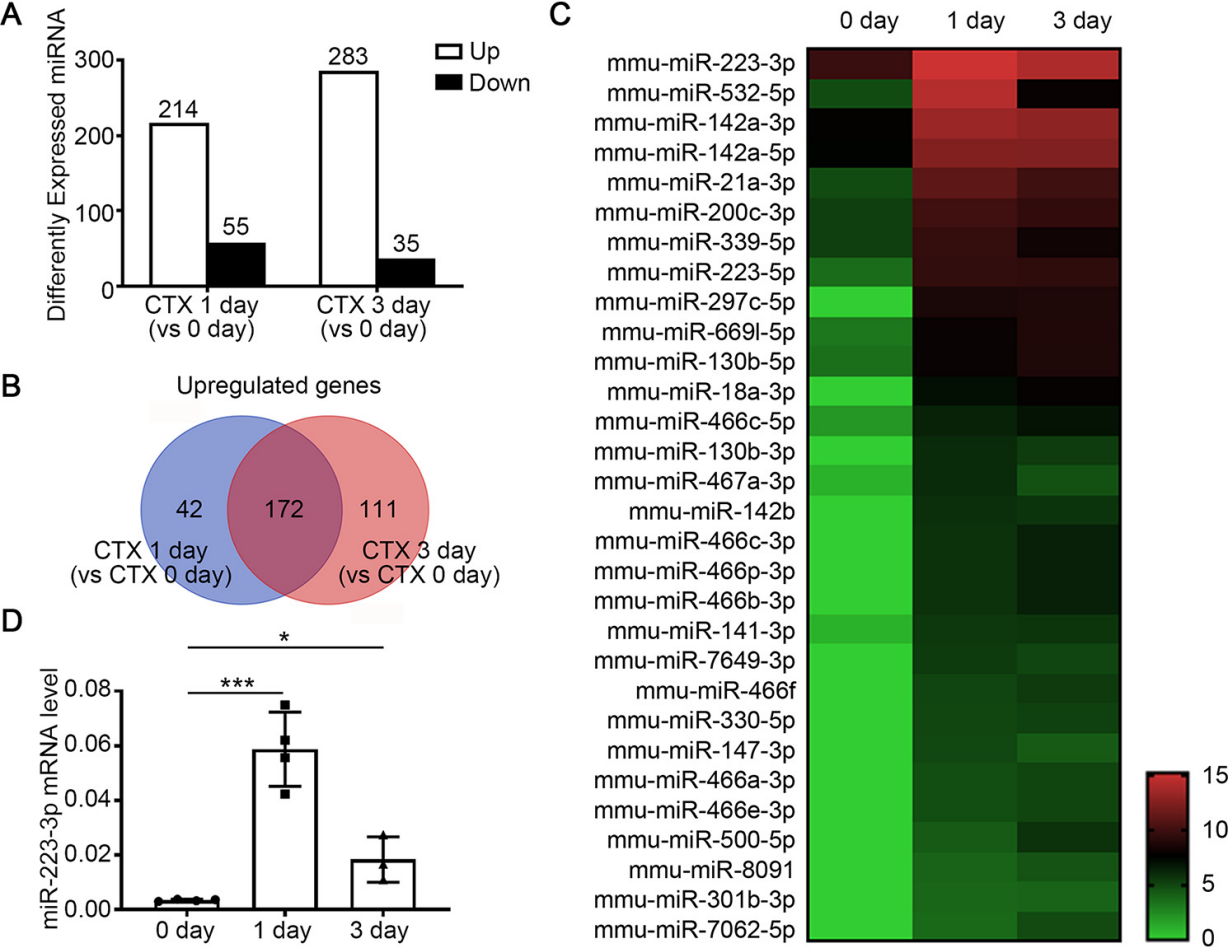
**MiR-223-3p is upregulated in skeletal muscle in the early stages of regeneration after injury.** *A–C*, miRNA expression profiling in the TA muscles of WT mice at different time points post-CTX injury by RNA-Seq. *A*, numbers of differentially expressed miRNAs with FC ≥ 2 and FDR ≤ 0.001 at 1 day and 3 days after injury compared with day 0. *B*, Wayne chart of the number of upregulated miRNAs at 1 day and 3 days after injury. *C*, heat map of selected differently expressed miRNAs at 0 day, 1 day, and 3 days after CTX injury. Each point reveals the log_2_ FPKM value of the indicated miRNA. *D*, RT-PCR analysis of miR-223-3p levels in WT TA muscles at 0 day, 1 day, and 3 days after injury (*n* = 3–4 per group). Data are expressed as the mean ± S.D. *, *p* < 0.05; ***, *p* < 0.001 by unpaired two-tailed Student's *t* test.

To identify novel miRNAs involved in the regulation of the inflammatory microenvironment during skeletal muscle regeneration, we intramuscularly injected WT mice with cardiotoxin (CTX) to generate an acute skeletal muscle injury and regeneration model. miRNA expression levels in the tibialis anterior (TA) muscles at days 1 and 3 post-CTX injury were compared with the levels in uninjured muscles (day 0) by miRNA-Seq analysis. There were 384 differentially expressed miRNAs either 1 or 3 days after injury (defined by fold change [FC] *versus* 0 day of ≥2 and false discovery rate [FDR] of ≤0.001), including 214 upregulated miRNAs and 55 downregulated miRNAs on day 1 and 283 upregulated miRNAs and 35 downregulated miRNAs on day 3 (Fig. 1*A*). 172 miRNAs were upregulated at both 1 day and 3 days (Fig. 1*B*).

We sequenced the 172 genes based on FC of 1 day compared with 0 day. Among the top 30 upregulated miRNAs, miR-223-3p was the most abundant miRNA on day 1 (Fig. 1*C*, Table S1). Using RT-PCR, we confirmed that miR-223-3p was significantly increased 1 day after muscle injury and decreased on day 3 but still significantly higher than its baseline level (Fig. 1*D*). These results validated the RNA-seq results, suggesting that miR-223-3p plays a role in the early inflammatory stage of skeletal muscle regeneration.

**Figure 2.**
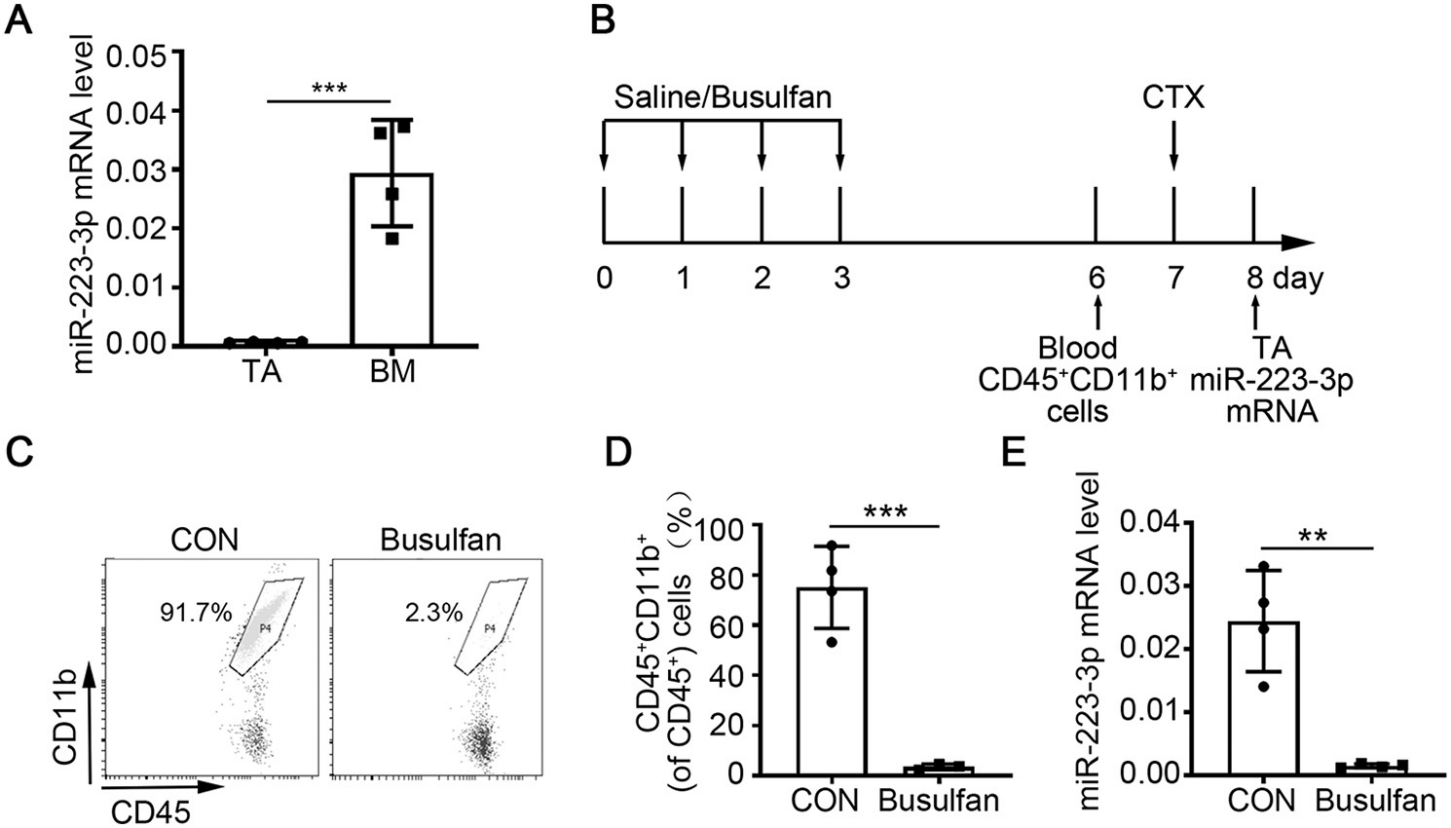
**Increased miR-223-3p is derived from myeloid mononuclear cells.** *A*, RT-PCR assessment of miR-223-3p levels in BM and muscle tissues from WT mice (*n* = 4 per group). *B*, schematic of the experimental design for the depletion of circulating myeloid-derived mononuclear cells by busulfan treatment. *C*, representative flow cytometry analysis of CD45^+^ CD11b^+^ cells from the peripheral blood of control and busulfan-treated mice. *D*, percentages of CD45^+^ CD11b^+^ cells among CD45^+^ cells in the peripheral blood of control and busulfan-treated mice (*n* = 3–4 per group). *E*, RT-PCR verification of miR-223-3p levels in the muscles of control and busulfan-treated mice at 1 day after CTX injury (*n* = 4 per group). Data are expressed as the mean ± S.D. **, *p* < 0.01; ***, *p* < 0.001 by unpaired two-tailed Student's *t* test.

At early stages of muscle injury and regeneration, the main cell components of muscle were muscle-resident cells and bone marrow (BM)-derived leukocytes. Therefore, to determine the mainly cellular source of increased miR-223-3p, we examined the expression levels of miR-223-3p in normal muscle tissue and BM tissue. The expression level of miR-223-3p was significantly higher in the BM tissue than in muscle (Fig. 2*A*). To further confirm the contribution of BM-derived cells to the upregulation of miR-223-3p, WT mice were injected with busulfan intraperitoneally to reduce the amounts of BM-derived mononuclear cells (CD45^+^ CD11b^+^) in the peripheral blood, and the expression of miR-223-3p was measured at 1 day after CTX injury by RT-PCR (Fig. 2*B*–*D*). As shown in Fig. 2*E*, the expression level of miR-223-3p increased in muscles from the untreated control group at 1 day after injury but did not increase in muscles from mice treated with busulfan. These results demonstrate that the increased miR-223-3p level in injured skeletal muscle is mainly derived from infiltrating BM-derived mononuclear cells.

### miR-223-3p deficiency impairs skeletal muscle regeneration

**Figure 3.**
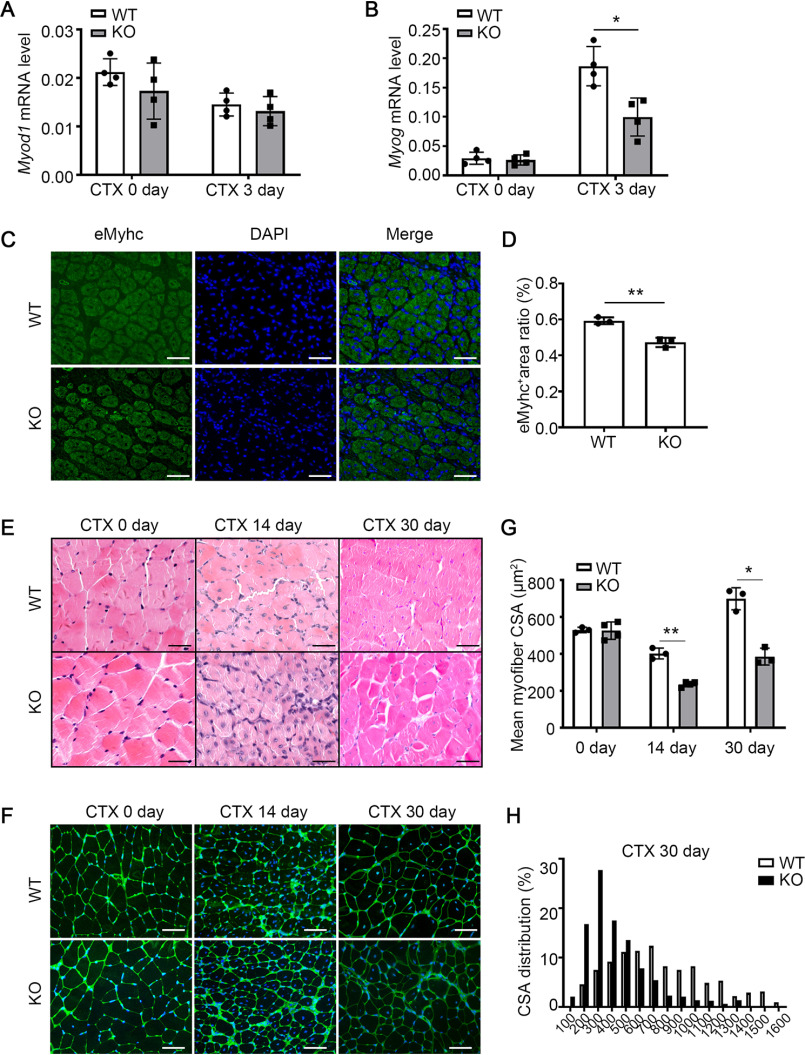
**miR-223-3p KO impairs skeletal muscle regeneration after injury.** *A–B*, RT-PCR assessment of *Myod1* and *Myog* expression in the muscles of WT and miR-223-3p KO mice on 0 day and 3 days after CTX injury (*n* = 4 per group). *C*, representative eMHC immunofluorescence (*green*) of muscles of WT and miR-223-3p KO mice at 7 days after CTX injury. The nuclei were counterstained with DAPI (*blue*). *Scale bar*, 50 μm. *D*, percentages of eMHC-positive areas in fields of view from muscles of WT and miR-223-3p KO mice at 7 days after CTX injury (*n* = 3 per group). *E*, representative HE staining of muscles of WT and miR-223-3p KO mice at 0 day, 14 days, and 30 days after CTX injury. *Scale bar*, 50 μm. *F*, representative WGA staining (*green*) of muscles of WT and miR-223-3p KO mice at 0 day, 14 days, and 30 days after CTX injury. The nuclei were counterstained with DAPI (*blue*). *Scale bar*, 50 μm. *G*, mean myofiber CSA of muscles of WT and miR-223-3p KO mice at 0 day, 14 days, and 30 days after CTX injury (*n* = 3–4 per group; for each sample, ≥300 myofibers were measured). *H*, CSA distributions of muscles of WT and miR-223-3p KO mice at 30 days after CTX injury. Data are expressed as the mean ± S.D. *, *p* < 0.05; **, *p* < 0.01 by unpaired two-tailed Student's *t* test.

To clarify the role of miR-223-3p in muscle regeneration, miR-223-3p knockout (KO) mice and WT mice were used to examine the phenotype-associated muscle regeneration. At baseline level, the body weight and mass ratio of TA or gastrocnemius (GAS) muscle to the tibia length of miR-223-3p KO mice were similar to those of WT mice (Fig. S1*A* and *B*). There was almost no miR-223-3p detected in miR-223-3p KO muscles at 0 day and 3 days after injury (Fig. S1*C* and *D*). Hematoxylin and eosin (HE) staining data revealed no differences in the distribution of myofiber cross-sectional area (CSA) between uninjured muscles from miR-223-3p KO and WT mice (Fig. S1*E* and *F*). We then detected the mRNA expression levels of myogenic differentiation 1 (*Myod1*) and myogenin (*Myog*), which are associated with MuSC proliferation and differentiation, and observed decreased expression of *Myog* in the muscles of miR-223-3p KO mice compared with that in WT mouse muscles at 3 days after injury but no significant difference in *Myod1* expression (Fig. 3*A*–*B*). At 7 days after injury, the positive areas of eMyHC in miR-223-3p KO muscles were smaller than those in WT muscles (Fig. 3*C* and *D*). At 14 days and 30 days after injury, the mean CSA of newly formed myofibers (indicated by central nuclei) from miR-223-3p KO muscles was smaller than that of WT muscles (Fig. 3*E*–*G*), and the distribution of myofiber CSA was still shifted forward to the left in miR-223-3p KO muscles, which indicated increased smaller myofibers and reduced larger myofibers (Fig. 3*H*). These results indicate that miR-223-3p deficiency impairs muscle regeneration.

### miR-223-3p deficiency promotes interstitial fibrosis in skeletal muscle after injury

**Figure 4.**
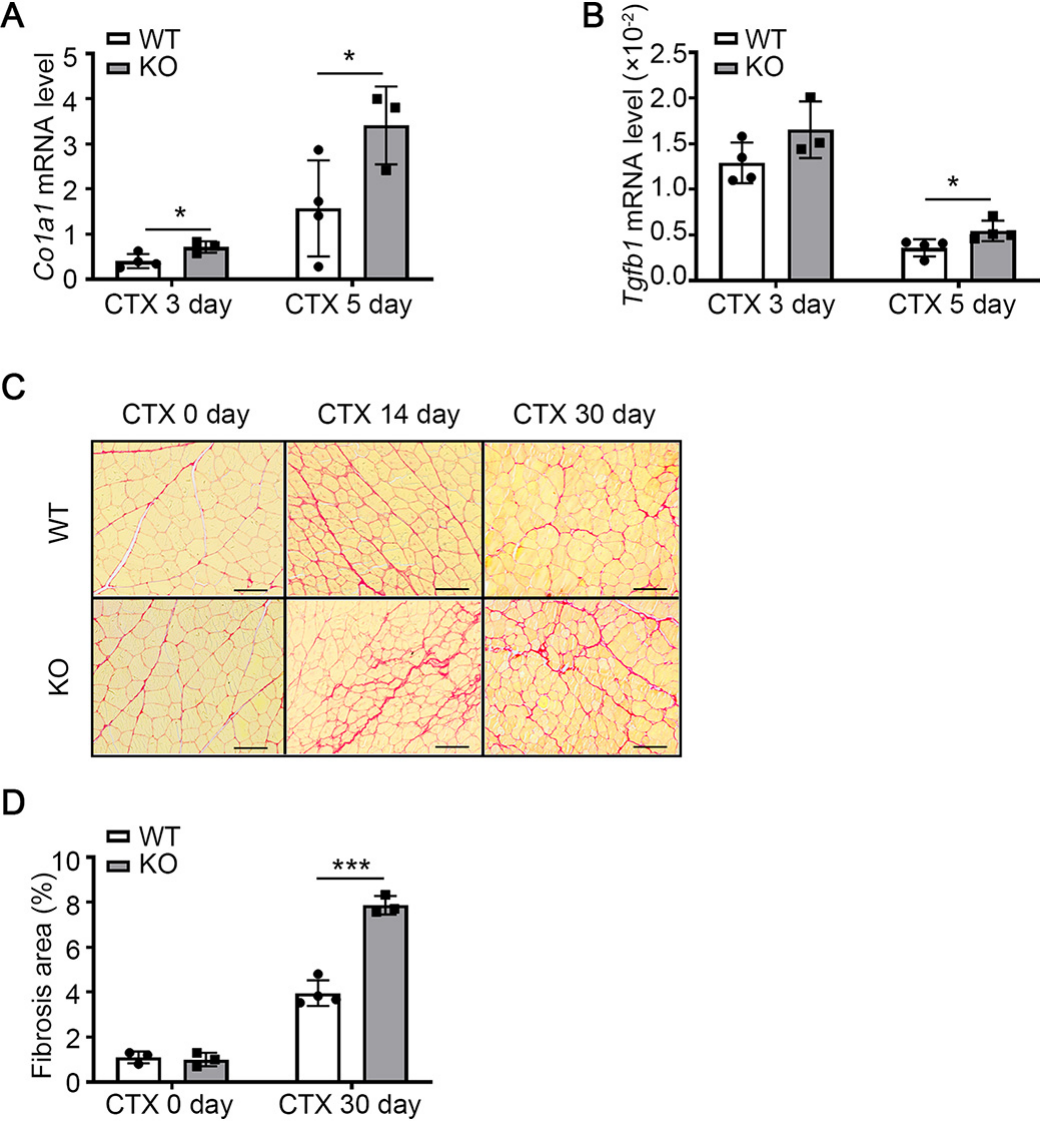
**miR-223-3p KO promotes interstitial fibrosis in injured skeletal muscle.** *A–B*, RT-PCR assessment of *Col1a1* and *Tgfb1* expression in the muscles of WT and miR-223-3p KO mice 3 days and 5 days after CTX injury (*n* = 3–4 per group). *C*, representative Picrosirius red staining (*red*) of muscles of WT and miR-223-3p KO mice on 0 day and 30 days after CTX injury. *Scale bar*, 100 μm. *D*, percentages of Picrosirius red-positive areas per field in the muscles of WT and miR-223-3p KO mice on 0 day and 14 days after CTX injury (*n* = 3–4 per group). Data are expressed as the mean ± S.D. *, *p* < 0.05; ***, *p* < 0.001 by unpaired two-tailed Student's *t* test.

To examine whether miR-223-3p affects the interstitial fibrosis formation that accompanies impaired muscle regeneration after injury, we used RT-PCR to measure the expression of collagen type I alpha 1 (*Col1a1*) and transforming growth factor beta 1 (*Tgfb1*), which are associated with fibrosis formation. *Col1a1* expression was significantly higher in miR-223-3p KO mice than in WT mice at 3 and 5 days after injury (Fig. 4*A*), and *Tgfb1* expression was significantly higher in miR-223-3p KO mice than in WT mice at 5 days after injury (Fig. 4*B*). We also examined collagen deposition in WT and miR-223-3p KO mouse muscles at 0, 14, and 30 days after injury by Pircosirius red staining. At baseline level, there was no difference in the ratio of interstitial fibrosis between WT and miR-223-3p KO muscle. However, at 30 days after injury, there was still more interstitial fibrosis formation in miR-223-3p KO muscles than in WT muscles (Fig. 4*C* and *D*). These data indicate that miR-223-3p deficiency promotes interstitial fibrosis in skeletal muscle after injury.

### Overexpression of miR-223-3p in WT mice does not affect skeletal muscle regeneration

To observe the effect of miR-223-3p overexpression on muscle regeneration, we administered WT mice with miR-223-3p agomir, because miRNA agomir has higher stability and miRNA activity *in vivo* than an miRNA mimic (Fig. S2*A*). At 3 days after injury, the expression of miR-223-3p in muscles from miR-223-3p agomir-treated mice was significantly higher than that of NC (negative control) agomir-treated mice, which indicated the overexpression efficiency of miR-223-3p agomir (Fig. S2*B*). At 30 days after CTX injury, muscle regeneration and fibrosis were examined. The mean myofiber CSA of muscles from miR-223-3p agomir-treated mice was similar to that of muscles from NC agomir-treated mice (Fig. S2*C–E*), and there was also no difference in the distribution of myofiber CSA in NC and miR-223-3p agomir-treated mice (Fig. S2*F*). In addition, the ratio of fibrosis area was not affected by miR-223-3p overexpression (Fig. S2*G* and *H*). These data indicated that overexpression of miR-223-3p in WT muscle did not affect muscle regeneration after injury.

### miR-223-5p is not critical for skeletal muscle regeneration after injury

Both miR-223-3p and miR-223-5p were upregulated in regenerating muscle. However, the fragments per kilobase of exon model per million reads mapped (FPKM) of miR-223-5p was lower than that of miR-223-3p at each time point (Table S1). To compare the effect of miR-223-3p and miR-223-5p in muscle regeneration, we also examined the function of miR-223-5p in muscle regeneration by inhibiting miR-223-5p in WT mice through miR-223-5p antagomir intravenous injection (Fig. S3*A*). At 3 days after injury, the expression of miR-223-5p in muscles from miR-223-5p antagomir-treated mice was significantly lower than that of NC antagomir-treated mice, which indicated the suppression efficiency of miR-223-5p antagomir (Fig. S3*B*). At 30 days after CTX injury, muscle regeneration and fibrosis were examined. The mean myofiber CSA of muscles from miR-223-5p antagomir-treated mice was similar to that of muscles from NC antagomir-treated mice (Fig. S3*C–E*), and there was also no difference in the distribution of myofiber CSA in NC and miR-223-5p antagomir-treated mice (Fig. S3*F*). The muscle fibrosis area ratio also was not affected by miR-223-5p inhibition (Fig. S3*G* and *H*). These data indicated that miR-223-5p was not critical for muscle regeneration after injury.

### miR-223-3p deficiency increases inflammation after skeletal muscle injury

The observed impaired muscle regeneration after injury in miR-223-3p KO mice prompted us to explore whether the proliferation and differentiation of MuSCs are affected by miR-223-3p. Primary MuSCs were isolated from WT mice and transfected with either an miR-223-3p mimic or a negative control (Fig. S4*A*). We observed similar *Myog* expression levels in primary MuSCs in each group after 3 days in differentiation medium (Fig. S4*B*). To detect the effect of miR-223-3p on MuSC proliferation, we performed a 5-ethynyl-2′-deoxyuridine (EdU) assay and analyzed the ratio of EdU-positive cells to total cells. However, the results showed that overexpression of miR-223-3p by miR-223-3p mimic does not affect the proliferation of MuSCs (Fig. S4*C* and *D*). To detect the effect of miR-223-3p on MuSC differentiation, we cultured MuSCs in differentiation medium for 3 days and observed the positive area ratio of embryonic isoform of myosin heavy chain (eMHC) by immunofluorescence. The results suggested that miR-223-3p is dispensable for MuSC differentiation (Fig. S4*E* and *F*). Taken together, these results indicate that miR-223-3p does not affect MuSC proliferation or differentiation.

**Figure 5.**
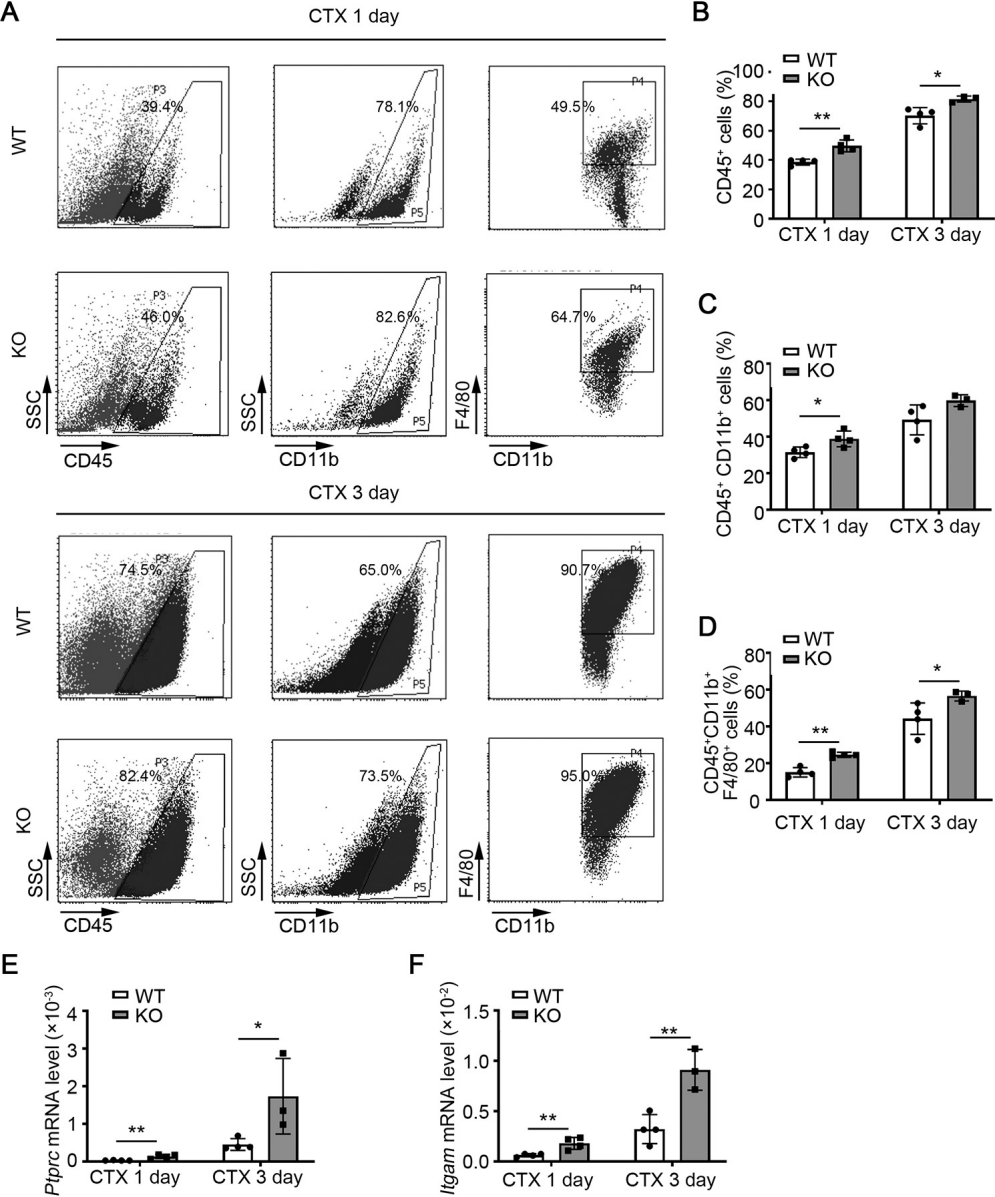
**miR-223-3p KO increases the inflammatory response in injured skeletal muscle.** *A*, representative flow cytometry analysis of CD45^+^ leukocytes, CD45^+^ CD11b^+^ monocytes, and CD45^+^ CD11b^+^ F4/80^+^ macrophages in the muscles of WT and miR-223-3p KO mice at 1 day and 3 days after CTX injury. *B*–*D*, percentages of CD45^+^, CD45^+^ CD11b^+^, and CD45^+^ CD11b^+^ F4/80^+^ cells in the muscles of WT and miR-223-3p KO mice at 1 day and 3 days after CTX injury (*n* = 3–4 per group). *E–F*, RT-PCR assessment of *Ptprc* and *Itgam* expression in the muscles of WT and miR-223-3p KO mice at 1 day and 3 days after CTX injury (*n* = 3–4 per group). Data are expressed as the mean ± S.D. *, *p* < 0.05; **, *p* < 0.01 by unpaired two-tailed Student's *t* test.

To determine whether miR-223-3p deficiency leads to an altered inflammatory response in skeletal muscle at early stages of regeneration following injury, we used flow cytometry to analyze the infiltration of inflammatory cells into injured muscle tissues from WT and miR-223-3p KO mice. There were more CD45^+^ leukocytes, CD45^+^ CD11b^+^ mononuclear cells, and CD45^+^ CD11b^+^ F4/80^+^ macrophages in miR-223-3p KO mouse muscles than in WT mouse muscles at 1 and 3 days after injury (Fig. 5*A*–*D*). The mRNA levels of protein tyrosine phosphatase receptor type C (*Ptprc-*encoded protein CD45) and integrin subunit alpha M (*Itgam*-encoded protein CD11b) were also significantly higher in miR-223-3p KO mouse muscles than that in WT muscles (Fig. 5*E* and *F*). These results suggest that loss of miR-223-3p results in a continuous inflammatory response in injured muscle.

### miR-223-3p deficiency leads to increased proinflammatory macrophage infiltration in injured skeletal muscle

**Figure 6.**
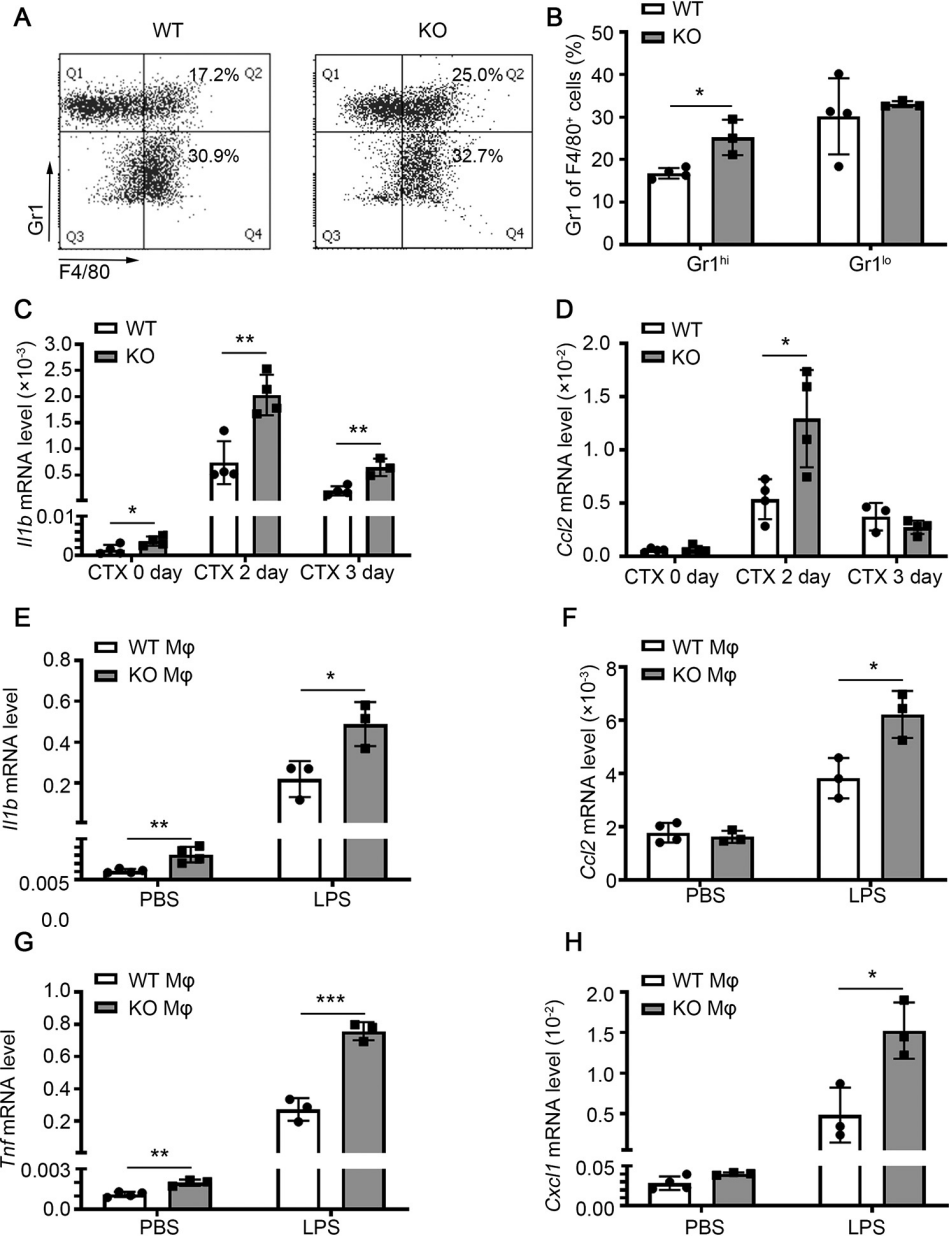
**miR-223-3p KO increases proinflammatory macrophage infiltration of injured skeletal muscle.** *A*, representative flow cytometry analysis of CD45^+^ CD11b^+^ F4/80^+^ Gr1^hi^ proinflammatory macrophages and CD45^+^ CD11b^+^ F4/80^+^ Gr1^lo^ anti-inflammatory macrophages in the muscle total macrophages of WT and miR-223-3p KO mice 1 day after CTX injury. *B*, percentages of CD45^+^ CD11b^+^ F4/80^+^ Gr1^hi^ macrophages and CD45^+^ CD11b^+^ F4/80^+^ Gr1^lo^ anti-inflammatory macrophages in the muscle total macrophages of WT and miR-223-3p KO mice at 1 day after CTX injury (*n* = 3–4 per group). *C*–*D*, RT-PCR assessment of *Il1b* and *Ccl2* expression in the muscles of WT and miR-223-3p KO mice at 0, 2, and 3 days after CTX injury (*n* = 3–4 per group). *E–H*, RT-PCR assessment of *Il1b*, *Ccl2*, *Tnf*, and *Cxcl1* expression in WT and miR-223-3p KO primary macrophages (mφ) treated with PBS or LPS (5 μg/ml) for 12 h (*n* = 3–4 per group). Data are expressed as the mean ± S.D. *, *p* < 0.05; **, *p* < 0.01; ***, *p* < 0.001 by unpaired two-tailed Student's *t* test.

To further explore the cause of the continuous inflammatory response caused by miR-223-3p deficiency, we analyzed the subtypes of infiltrating macrophages at 1 day after injury, using Gr1 to distinguish pro- and anti-inflammatory macrophages. The ratio of F4/80^+^ Gr1^hi^ proinflammatory macrophages in macrophages of miR-223-3p KO muscles was higher than that in WT muscle, whereas there was no significant difference in the ratio of F4/80^+^ Gr1^lo^ anti-inflammatory macrophages in these samples (Fig. 6*A* and *B*). Moreover, the mRNA level of the proinflammatory cytokine *Il1b* was significantly increased in miR-223-3p KO muscles at days 0, 2, and 3 after injury compared with that in the WT muscles, and the expression of *Ccl2* was also significantly higher in miR-223-3p KO muscles than in WT muscles at 2 days after injury (Fig. 6*C* and *D*). *In vitro*, activated miR-223-3p KO macrophages (stimulated with lipopolysaccharide [LPS] for 12 h) expressed higher mRNA levels of *Il1b* and tumor necrosis factor (*Tnf*), *Ccl2*, and C-X-C motif chemokine ligand 1 (*Cxcl1*) than WT macrophages, whereas there was no significant difference in baseline levels when the cells were treated with PBS (Fig. 6*E*–*H*).

Collectively, these results indicate that at early stages in skeletal muscle regeneration after injury, miR-223-3p KO mice display an imbalance in pro- and anti-inflammatory factors in their microenvironment, reflected by increased macrophage infiltration and proinflammatory macrophage differentiation.

### IL-6 is the target gene of miR-223-3p during muscle regeneration

**Figure 7.**
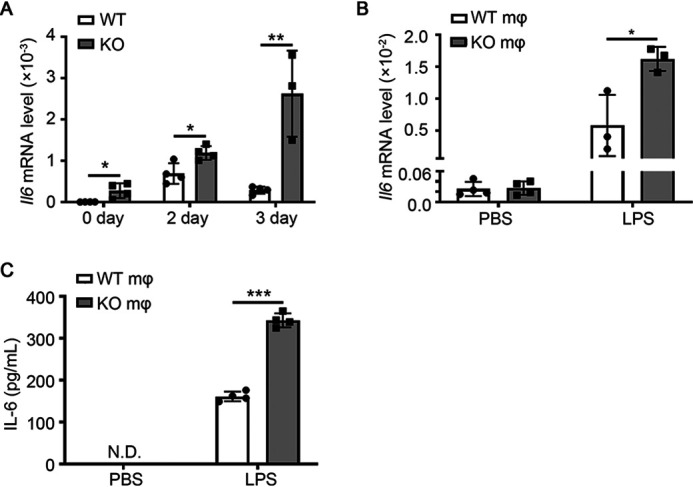
**miR-223-3p targets *Il6* during muscle regeneration.** *A*, RT-PCR assessment of *Il6* expression in the muscles of WT and miR-223-3p KO mice at 0, 2, and 3 days after CTX injury (*n* = 3–4 per group). *B*, RT-PCR assessment of *Il6* expression in WT and miR-223-3p KO primary macrophages treated with PBS or LPS (5 μg/ml) for 12 h (*n* = 3–4 per group). *C*, cytometric bead array assessment of the protein concentration of IL-6 in the culture medium of WT or miR-223-3p KO primary macrophages treated with PBS or LPS (5 μg/ml) for 12 h (*n* = 4 per group). Data are expressed as the mean ± S.D. *, *p* < 0.05; **, *p* < 0.01; ***, *p* < 0.001, by unpaired two-tailed Student's *t* test.

Previous studies have reported that miR-223-3p inhibits proinflammatory responses in the liver and lungs by directly targeting *Il6* (15, 16). In addition, our previous study demonstrated that the IL-6 signal transducer and activator of transcription 3 (STAT3) pathway promotes macrophage infiltration during muscle regeneration (8). To determine whether *Il6* is involved in the increased proinflammatory macrophage infiltration observed in miR-223-3p KO skeletal muscle, RT-PCR was performed. The results reveal significant *Il6* upregulation in miR-223-3p KO skeletal muscles compared with that in WT muscles at 0, 2, and 3 days after injury (Fig. 7*A*). *In vitro*, activated miR-223-3p KO macrophages (stimulated with LPS for 12 h) expressed higher levels of *Il6* mRNA than WT macrophages, whereas there was no significant difference in baseline *Il6* levels when the cells were treated with PBS. The concentration of IL-6 was increased in the culture medium of activated miR-223-3p KO macrophages compared with that of activated WT macrophages; however, baseline IL-6 levels were undetectable (Fig. 7*B* and *C*). These results suggest that IL-6 is the target gene of miR-223-3p during muscle regeneration.

### Administration of IL-6-neutralizing antibody rescues the muscle regeneration ability of miR-223-3p KO mice

**Figure 8.**
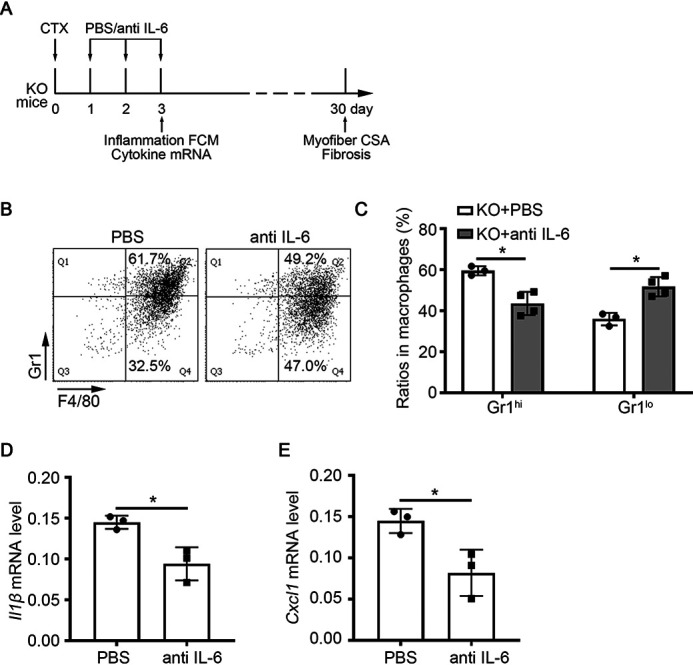
**IL-6-neutralizing antibody reduced the proinflammatory response in miR-223-3p KO muscle.** *A*, schematic of the experimental design. IL-6-neutralizing antibody (10 μg/TA) or PBS was intramuscularly injected into WT or miR-223-3p KO muscle at 1 day, 2 days, and 3 days after CTX injury. At day 3 after injury, TA muscle was harvested to examine the inflammation response, and at day 30 after injury, TA muscle was harvested to examine the regeneration. *B*, representative flow cytometry analysis of CD11b^+^ F4/80^+^ Gr1^hi^ proinflammatory macrophages and CD11b^+^ F4/80^+^ Gr1^lo^ anti-inflammatory macrophages in the muscle total macrophages of miR-223-3p KO or miR-223-3p KO muscle with IL-6-neutralizing antibody at 3 days after CTX injury. *C*, percentages of CD11b^+^ F4/80^+^ Gr1^hi^ macrophages and CD45^+^ CD11b^+^ F4/80^+^ Gr1^lo^ anti-inflammatory macrophages in the muscle total macrophages of miR-223-3p KO or miR-223-3p KO muscle with IL-6-neutralizing antibody after CTX injury (*n* = 3–4 per group). *D–E*, RT-PCR assessment of *Il1b* and *Cxcl1* expression in muscles at 3 days after CTX injury (*n* = 3 per group). Data are expressed as the mean ± S.D. *, *p* < 0.05 by unpaired two-tailed Student's *t* test.

**Figure 9.**
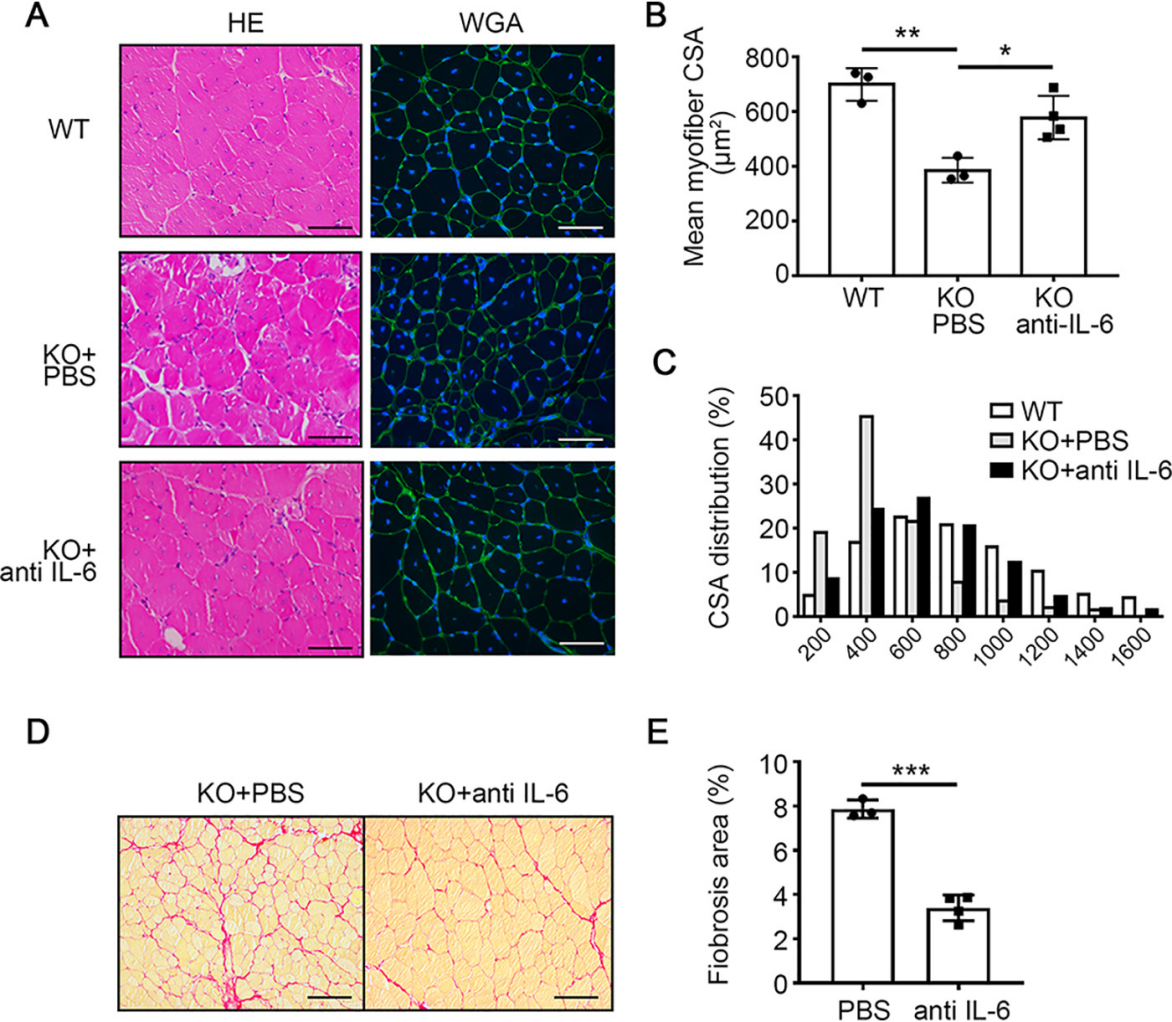
**IL-6-neutralizing antibody rescued the impaired muscle regeneration of miR-223-3p KO mice.** *A*, representative HE (*left*) and WGA staining (*green*, *right*) of WT, miR-223-3p KO, or miR-223-3p KO muscle with IL-6-neutralizing antibody at 30 days after CTX injury. The nuclei were counterstained with DAPI (*blue*). *Scale bar*, 50 μm. *B*, mean myofiber CSA of WT, miR-223-3p KO, or miR-223-3p KO muscle with IL-6-neutralizing antibody at 30 days after CTX injury (*n* = 3–4 per group; for each sample, ≥300 myofibers were measured). *C*, CSA distributions of WT, miR-223-3p KO, or miR-223-3p KO muscle with IL-6-neutralizing antibody at 30 days after CTX injury. *D*, representative Picrosirius red staining (*red*) of miR-223-3p KO or miR-223-3p KO muscles with IL-6-neutralizing antibody at 30 days after CTX injury. *Scale bar*, 100 μm. *E*, percentages of Picrosirius red-positive areas per field in miR-223-3p KO or miR-223-3p KO muscles with IL-6-neutralizing antibody at 30 days after CTX injury (*n* = 3–4 per group). Data are expressed as the mean ± S.D. *, *p* < 0.05; **, *p* < 0.01; ***, *p* < 0.001 by unpaired two-tailed Student's *t* test.

To examine whether the excessive IL-6 was related to the increased inflammation and impaired muscle regeneration in miR-223-3p KO muscles, the IL-6-neutralizing antibody or PBS was intramuscularly injected into miR-223-3p KO mice at 1, 2, and 3 days after CTX injury (Fig. 8*A*). At 3 days after injury, the ratio of F4/80^+^ Gr1^hi^ proinflammatory macrophages was decreased in macrophages of miR-223-3p KO muscles with IL-6-neutralizing antibody, whereas the ratio of F4/80^+^ Gr1^lo^ anti-inflammatory macrophages was increased in macrophages of miR-223-3p KO muscles with IL-6-neutralizing antibody (Fig. 8*B* and *C*). The mRNA expression levels of the proinflammatory cytokines *Il1*β and *Cxcl1* were also decreased in miR-223-3p KO muscles with IL-6-neutralizing antibody (Fig. 8*D* and *E*). At 30 days after injury, the muscle regeneration and fibrosis were examined. IL-6-neutralizing antibody could increase the mean myofiber CSA of miR-223-3p KO muscles (Fig. 9*A* and *B*) and the distribution of myofiber CSA shifted forward to larger areas (Fig. 9*C*). The fibrosis area in miR-223-3p KO muscles was also reduced by the administration of IL-6-neutralizing antibody (Fig. 9*D* and *E*). These data demonstrated that increased IL-6 expression drives the inflammatory imbalance observed in miR-223-3p-deficient injured muscle.

## Discussion

Our results demonstrate that miR-223-3p plays an important role in regulating inflammation and muscle regeneration after injury. miR-223-3p deficiency led to continuously increased inflammation in injured muscle and impaired muscle regeneration. Mechanistically, miR-223-3p deficiency in macrophages is linked to increased expression of target gene *Il6*.

Data from our miRNA transcriptome analysis showed that miR-223-3p was significantly increased in injured muscle in the early stage of regeneration. A previous study of Chen *et al*. also examined the miRNA expression profile by using Taqman miRNA expression assays (17). In this study, we detected the miRNA expression profile by using small RNA-Seq. Small RNA sequencing could detect the expression of miRNA at a higher throughput, and we identified more differentially expressed miRNA at each time point. Although the exact fold change of miR-223-3p at day 1 and day 3 after injury was different, both our and their data showed a similar change trend in the miR-223-3p expression level. However, the previous study did not focus on this and the roles of miRNA in muscle regeneration. miR-223-3p is mainly expressed in myeloid cells (18–21), and its expression is associated with changes in the numbers of infiltrating myeloid cells (2, 5, 17). In our study, removing circulating BM-derived mononuclear cells by busulfan treatment (22, 23) attenuated the upregulation of miR-223-3p after injury revealed that the increased miR-223-3p in injured muscles was mainly derived from recruited BM mononuclear cells. BM-derived cells have been identified as the source of elevated miR-223-3p levels in other disease models as well (19–21).

Our study identifies miR-223-3p as a critical component of muscle regeneration for the first time. Skeletal muscle regeneration after injury mainly relies on the activation, proliferation, and differentiation of MuSCs to form new myofibers, and the inflammatory microenvironment is critical to fine-tune this process (2, 5–7, 24). In this study, we observed that during muscle regeneration after injury, the expression of the differentiation-associated gene *Myog* was decreased in miR-223-3p KO mouse muscles. Previous studies have shown that miR-223-3p directly promotes differentiation and inhibits proliferation in chicken embryonic MuSCs (25). However, an miR-223-3p mimic had no effect on the proliferation or differentiation of mouse primary MuSCs. This could be because of differences in the processes of embryonic muscle development and mature skeletal muscle regeneration.

miR-223-3p participates in a negative feedback mechanism, limiting excessive inflammation and maintaining BM cell homeostasis (18, 26–29). In this study, we found that the number of leukocytes (particularly macrophages) and the expression of inflammatory factors were significantly increased in the early stage of regeneration after muscle injury in miR-223-3p KO mice. Therefore, the balance of pro- and anti-inflammatory factors in the microenvironment is disrupted in the skeletal muscle of these mice. A persistent or increased proinflammatory state can exacerbate tissue injury and may blunt the differentiation and fusion of MuSCs (30, 31).

Our results indicate that IL-6, a previously reported direct target of miR-223-3p, is involved in the inflammatory imbalance in miR-223-3p KO mouse muscle after injury. Previous studies have shown that miR-223-3p can directly bind the 3′ UTR of *Il6* and inhibit its expression during inflammatory responses after liver and lung diseases (15, 16). IL-6 is a pleiotropic cytokine, which is released by muscle fiber, MuSCs, FAP, and inflammatory cells under different conditions (10, 32). During muscle homeostasis, or physical exercise, muscle fibers produce and secrete IL-6 as a myokine, which has been considered an energy sensor modulating both lipolysis and glucose uptake to adapt to the energy requirements during exercise (33). The elevated IL-6 could also promote muscle hypertrophy by increasing protein synthesis and promoting the proliferation of satellite cells and their incorporation into the existing myofiber (34). Conversely, under pathologic conditions, such as aging and chronic inflammation disease-associated cachexia, the plasma levels of IL-6 increase persistently, promoting muscle atrophy primarily by inducing catabolic pathways (35, 36). The overexpression of IL-6 in transgenic mice caused muscular atrophy and increased levels of cathepsin in skeletal muscle, indicating that IL-6 is involved in the regulation of muscle protein degradation (37). A recently study shows that aberrantly activated IL-6-STAT3 signaling in FAPs results in impaired regeneration and increased fibrosis in denervated muscles (38).

After acute muscle injury, the infiltrated macrophage is the main cellular source, and the increased IL-6 could stimulate the proliferation of MuSCs and promote the expression of chemokines CCL2 and CCL3 in macrophages, which further enhances the infiltration of macrophages (8). At the late stage of muscle injury and regeneration, the expression of IL-6 returns to the basal level, which is accompanied with the resolution of inflammatory response. In this study, we found that as a target gene of miR-223-3p, the expression of IL-6 persisted at a higher level in miR-223-3p KO muscles, and the level of inflammatory cells in miR-223-3p KO muscles was higher than that in WT muscles. When the IL-6-neutralizing antibody was administered in miR-223-3p KO muscles, the numbers of proinflammatory macrophages and cytokines were reduced and the regeneration ability of miR-223-3p KO muscles was also restored. Our data also support the conclusion that the excessive increase of IL-6 in muscle was detrimental to skeletal muscle homeostasis and regeneration.

In this study, we found that overexpression of miR-223-3p in normal WT muscle did not affect muscle regeneration after injury. The expression of miR-223-3p was already abundant in normal WT muscle after injury, and the function of the overexpression of miR-223-3p in normal muscle could not be detected. Under some pathological conditions with cachexia, such as chronic kidney dysfunction, the expression of miR-223-3p was decreased (39). The function of miR-223-3p agomir in muscle regeneration should be further explored under these conditions.

In conclusion, we demonstrate that myeloid cell-derived miR-223-3p has a protective effect on muscle regeneration after injury, repressing a continuously proinflammatory response by repressing target gene *Il6*.

## Experimental procedures

### Animals

We purchased miR-223-3p KO mice in the C57BL/6 background from The Jackson Laboratory (stock no. 013198). miR-223-3p KO mice are viable and fertile and had body weight similar to that of littermate WT control mice. Mice were bred in the animal facility of Beijing Anzhen Hospital, where they were fed a standard diet and housed in a specific-pathogen-free environment with a 12-h/12-h light/dark cycle. 10–12-week-old male mice were used in this study. The Animal Subjects Committee of Capital Medical University approved all animal housing and experimental protocols.

### Muscle regeneration model

Mice were anesthetized with 2% isoflurane and maintained with 1.5% isoflurane (RWD Life Science, Shenzhen, China), and the TA and GAS muscles were injected with 10 μm CTX from *Naja pallida* (30 and 60 μl; Sigma, St. Louis, MO, USA) or saline as a control. At different time points after skeletal muscle injury, the mice were euthanized by intraperitoneal injection with sodium pentobarbital (100 mg · kg^−1^), and the TA and GAS muscles were harvested. Unless otherwise specified, TA tissues were fixed in 10% neutral buffered formalin for 8 h and stored in paraffin at room temperature for histological analysis. GAS tissues were frozen in liquid nitrogen and stored at −80 °C for mRNA extraction.

### Pretreatment with busulfan

Busulfan (Sigma) was intraperitoneally injected into mice (35 mg·kg^−1^·day^−1^) for 4 consecutive days. 6 days after the first injection, the efficiency of myeloid mononuclear macrophage and neutrophil removal from the peripheral blood was examined by flow cytometry. The TA muscles were injected with CTX 7 days after busulfan injection and harvested the next day for mRNA extraction.

### Histological analysis

TA muscles were sectioned to 5-μm thickness. Staining with HE and FITC-conjugated wheat germ agglutinin (WGA) (1:50; Sigma) to evaluate muscle CSA, Picrosirius red to detect collagen deposition, and eMHC (1:200; Developmental Studies Hybridoma Bank, Iowa City, IA, USA) to detect myofiber differentiation were performed as previously described (13, 40). Images were captured on an ECLIPSE 90i fluorescence digital microscope (Nikon, Japan) and analyzed using NIS-Elements Br 3.0 software (Nikon).

### RNA extraction and RT-PCR

Total RNA was extracted using TRIzol (Invitrogen, Carlsbad, CA, USA) as previously described (8). For miRNA, 2 μg RNA was reverse transcribed to cDNA using the TaqMan microRNA reverse transcription kit (Applied Biosystems, Lithuania) and primers targeting miR-223-3p/5p and U6 small nuclear RNA (TaqMan MicroRNA assay, Applied Biosystems). miRNA expression levels were measured by RT-PCR using TaqMan Universal Master Mix II (Applied Biosystems, Foster City, CA, USA) and probes targeting miR-223-3p/5p and U6 (TaqMan MicroRNA Assay, Applied Biosystems) in a CFX Connect real-time PCR detection system (Bio-Rad, USA). For mRNAs, cDNA was generated from 2 μg total RNA using the reverse transcription kit (Promega, Madison, WI, USA). The sequences of all cDNA primers are listed in Table S2. The expression levels of mRNAs were measured by RT-PCR using SYBR Master Mix II (Takara, Otsu, Shiga, Japan). Relative expression was calculated from cycle threshold values using the housekeeping gene β-actin (*Actb*; for mRNA) and U6 (for miRNA) as controls.

### miRNA sequencing

Four samples were pooled into one sample for miRNA sequencing at each time point. miRNA sequencing and analysis were performed on the BGISEQ platform at BGI (Shenzhen, China). In brief, after the quality and integrity of total RNA were assessed on an Agilent 2100 Bioanalyzer (Agilent Technologies, USA), small RNAs (18–30-nucleotide segments) were separated from total RNA by PAGE and processed for library construction and sequencing. High-quality clean read sequences were screened by alignment with National Center for Biotechnology Information data (GCF_000001635.26_GRCm38.p6). Afterward, FC and FDR-corrected *p* values were calculated. FDR ≤ 0.001 and FC ≥ 2 were used as thresholds to identify differentially expressed miRNAs.

### Flow cytometry analysis

TA muscles were dissected, finely minced, and dissociated by digestion with 200 units/ml collagenase I (Gibco, Grand Island, NY, USA) and 2.4 units/ml dispase II (Gibco) for 30 min at 37 °C and 100 rpm. The muscle slurry was diluted in PBS, filtered, centrifuged at 1,500 rpm for 5 min, and resuspended in PBS. Mononuclear cells (1 × 10^6^ in 100 μl PBS) were labeled with diluted CD45 peridinin chlorophyll protein Cy5.5, CD11b allophycocyanin Cy7, F4/80 allophycocyanin, and Gr1 phycoerythrin antibodies (all antibodies from BD, USA) for 30 min at 4 °C, and then cells were washed and resuspended in PBS buffer. Cells were analyzed by flow cytometry (BD LSRFortessa) and associated software (BD FACSDiva software).

### Isolation and culture of primary BMDMs

Primary BM-derived macrophages (BMDMs) were generated as previously described (41). In brief, BMDMs were maintained in growth medium (Dulbecco's modified Eagle's medium, high glucose, supplemented with 10% fetal bovine serum and 1% penicillin-streptomycin). After 4 h, nonadherent cells were removed, and adhered cells were induced to differentiate into macrophages with fresh growth medium supplemented with 50 ng/ml macrophage colony-stimulating factor (PeproTech, Rocky Hill, NJ, USA). Cells were cultured in a humidified incubator at 37 °C with 5% CO_2_. To activate BMDMs, cells were stimulated with 5 μg/ml LPS (PeproTech) for 12 h.

### Cytometric beads array assays

The concentration of IL-6 in culture medium was determined by a CBA protein assay kit IL-6 flex set (Thermo Fisher, Waltham, MA, USA). The CBA immunoassay was carried out according to the manufacturer's instructions.

### Treatment with IL-6-neutralizing antibody

IL-6-neutralizing antibody (5 μg/TA; RD, Minneapolis, MN, USA) or PBS as a control was intramuscularly injected into WT or miR-223-3p KO muscle at 1 day, 2 days, and 3 days after CTX injury, and mice were analyzed at the indicated time points.

### Statistical analysis

Unless otherwise stated, all data are expressed as the mean ± S.D. Statistical analysis was performed using GraphPad Prism 8.30 software, and the two groups were compared using unpaired two-tailed Student's *t* tests. *p* values of <0.05 were considered statistically significant.

## Data availability

miRNA sequencing data have been deposited in the Gene Expression Omnibus under accession number GSE141879. All other data are contained within the manuscript.

## Supplementary Material

Supporting Information
