# Supplementary material for: MicroRNA-223-3p promotes skeletal muscle regeneration by regulating inflammation in mice
Source: J Biol Chem. 2020 Jun 3;295(30):10212–23. doi: 10.1074/jbc.RA119.012263 (PMC7383371; doi:10.1074/jbc.RA119.012263)
Supplement: Supporting Information [file supp_295_30_10212__index.html]

MicroRNA-223-3p promotes skeletal muscle regeneration by regulating inflammation in mice — MiR-223-3p promotes muscle regeneration — MicroRNA-223-3p promotes skeletal muscle regeneration by regulating inflammation in mice — miR-223-3p promotes muscle regeneration — Supporting Information 

# MicroRNA-223-3p promotes skeletal muscle regeneration by regulating inflammation in mice

## Supporting Information

- Supporting Information (to be published online) - Supplemental experimental procedures, supplemental figure 1-4, supplemental table 1-2.
