# Supplementary material for: MicroRNA-223-3p promotes skeletal muscle regeneration by regulating inflammation in mice
Source: J Biol Chem. 2020 Jun 3;295(30):10212–23. doi: 10.1074/jbc.RA119.012263 (PMC7383371; doi:10.1074/jbc.RA119.012263)
Supplement: Supporting Information [file supp_RA119.012263_157640_2_supp_540780_qbpwk2.pdf]

MicroRNA-223-3p promotes skeletal muscle regeneration by regulating inflammation in mice

Naixuan Cheng[1, 2], Chang Liu[2], Yulin Li[2], Shijuan Gao[2], Ying-Chun Han [2], Xiaonan Wang[2, 3], Jie Du[2, *], Congcong Zhang[2, *]

- **Supplemental Experimental procedures**
- **Supplemental Figure 1-4**
- **Supplemental Table 1-2**

Experimental procedures

MiRNA agomir or antagomir in vivo transfection. miRNA agomir or antagomir (0.6μMol·Kg^{-1}, RiboBio, Guangzhou, China) was injected into WT mice angula veins at 3 day and 1 day before and after CTX injury and mice were analyzed at the indicated time points.

Primary MuSC isolation, culture, and differentiation Primary MuSCs were isolated from mice as previously described (40). In brief, hindlimb muscles were dissected, finely minced, and dissociated by digestion with collagenase I (200 U/mL, Gibco) and dispase II (2.4 U/mL, Gibco) for 45 minutes at 37°C and 100 rpm. The muscle slurry was diluted in PBS, filtered through a 100-μm cell strainer, and centrifuged at 1,500 rpm for 5 minutes. Cells were resuspended in growth culture medium (F-10 medium supplemented with 10% fetal bovine serum and 1% penicillin-streptomycin) and seeded in a dish. After two hours, unadhered cells were transferred to a Matrigel (Corning, Bedford, MA, USA)-coated dish. After 24 hours, unattached cells were removed, and the adherent cells (MuSCs) were cultured with growth medium in a humidified incubator at 37°C with 5% CO_2. For MuSC differentiation, cells were cultured in differentiation medium (F-10 medium supplemented with 2% horse serum and 1% penicillin-streptomycin) for 3 days.

MuSC proliferation and differentiation analysis Proliferation analysis was performed using the Cell Light EdU Apollo 488 In Vitro Kit (RiboBio) according to the manufacturer's instruction. In brief, 50 μM EdU was added to the growth medium of MuSCs. Four hours later, cells were fixed in 4% paraformaldehyde for 30 minutes, permeabilized with 0.5% Triton X-100 for 10 minutes, and incubated with Apollo staining reaction solution for 30 minutes at room temperature. Nuclei were counterstained with Hoechst in PBS. An ImageXpress Micro High-Content Imaging System (Molecular Devices, USA) was used to acquire images of random fields of view and analyze the percentage of EdU$^+$ MuSCs cells. For differentiation analysis, MuSCs were fixed in 4% paraformaldehyde for 10 minutes and incubated with primary antibody eMHC (1:200; Developmental Studies Hybridoma Bank) at 4°C overnight after permeabilizing with 0.5% Triton X-100 for 10 minutes and blocking with serum for 30 minutes, then incubated with Alexa 488-conjugated polyclonal anti-mouse IgG secondary antibody (1:1000; Cell Signaling Technologies, Carlsbad, CA, USA) at 37°C for 30 minutes and stained with 4′,6-diamidino-2-phenylindole (DAPI; Vector Labs, Burlingame, CA, USA). An ECLIPSE 90i fluorescence digital microscope was used to acquire images and NIS-Elements Br 3.0 software was used to analyze the

eMHC$^+$ area.

MiRNA mimic in vitro transfection transfection Primary MuSCs were transfected with micrON mmu-miR-223-3p mimic (50 nM) or a negative control mimic (50 nM; both RiboBio) using Lipofectamine 3000 (Invitrogen) according to the manufacturer's guidelines. Cells were harvested or treated 36 hours after transfection. The efficiency was confirmed by RT-PCR examining miRNA expression after transfection.

Figure S1

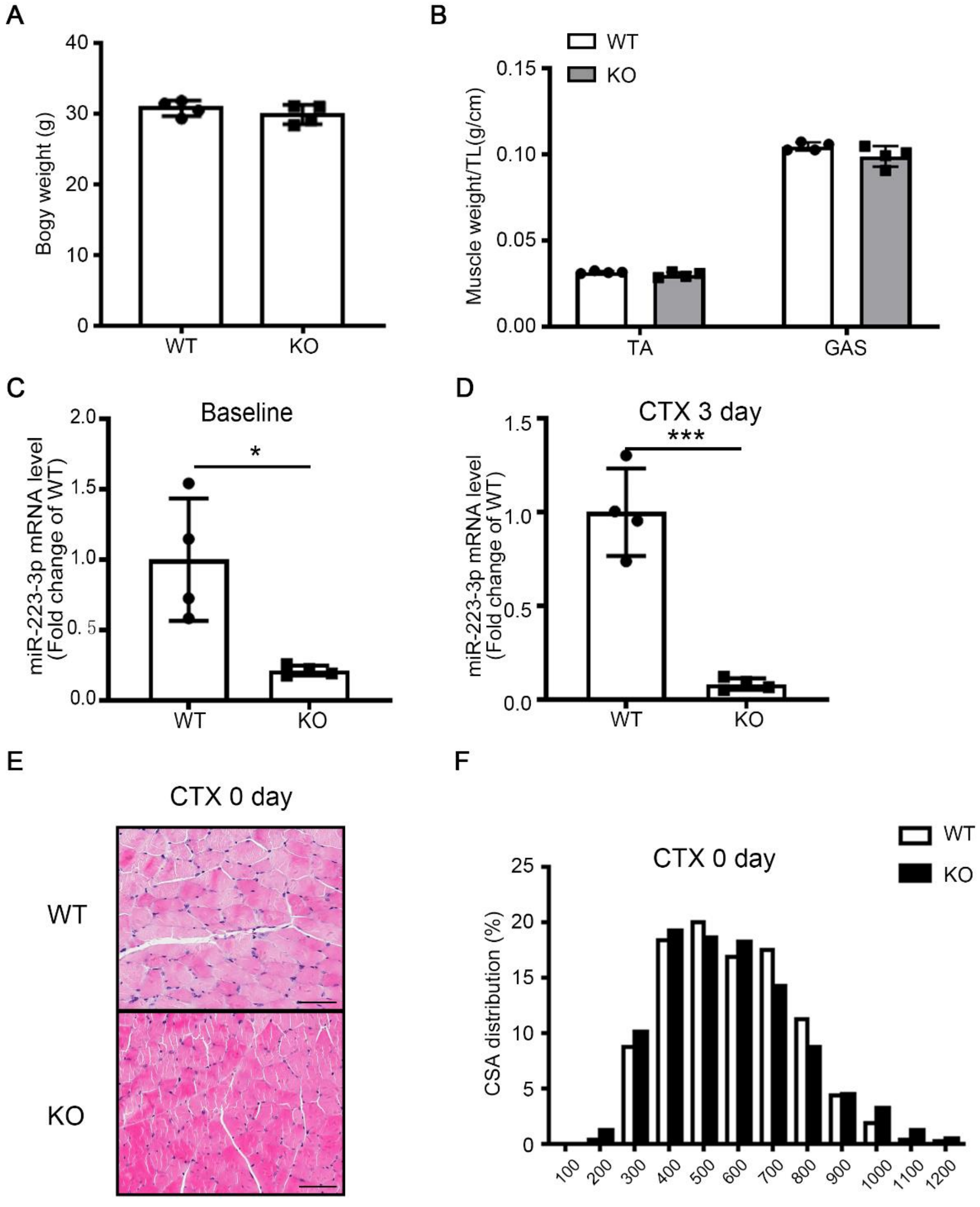
A
Bogy weight (g)
40
30
20
10
0
WT
KO
B
WT
KO
Muscle weight/TL(g/cm)
0.15
0.10
0.05
0.00
TA
GAS
C
Baseline
*
miR-223-3p mRNA level
(Fold change of WT)
2.0
1.5
1.0
0.5
0.0
WT
KO
D
CTX 3 day
***
miR-223-3p mRNA level
(Fold change of WT)
1.5
1.0
0.5
0.0
WT
KO
E
CTX 0 day
WT
KO
F
CTX 0 day
WT
KO
CSA distribution (%)
25
20
15
10
5
0
100
200
300
400
500
600
700
800
900
1000
1100
1200

Figure S1. General physiology of miR-223-3p KO mice

A. Body weight of WT and miR-223-3p KO mice at baseline (n=4 per group).

B. Ratio of TA mass to the tibia length of WT and miR-223-3p KO mice at baseline (n=4 per group).

C. RT-PCR assessment of miR-223-3p expression in uninjured muscles of WT and miR-223-3p KO mice (changed fold compared with WT muscle) (n=4 per group).

D. RT-PCR assessment of miR-223-3p expression in muscles of WT and miR-223-3p KO mice 3 days after injury (changed fold compared with WT muscle) (n=4 per group).

F. Representative HE staining of uninjured muscles of WT and miR-223-3p KO mice. Scale bar, 100 μm.

G. CSA distributions of uninjured muscles of WT and miR-223-3p KO mice.

Data are expressed as the mean ± SD. $^{*}P < 0.05$, $^{***}P < 0.001$ by unpaired two-tailed Student's t-test.

Figure S2

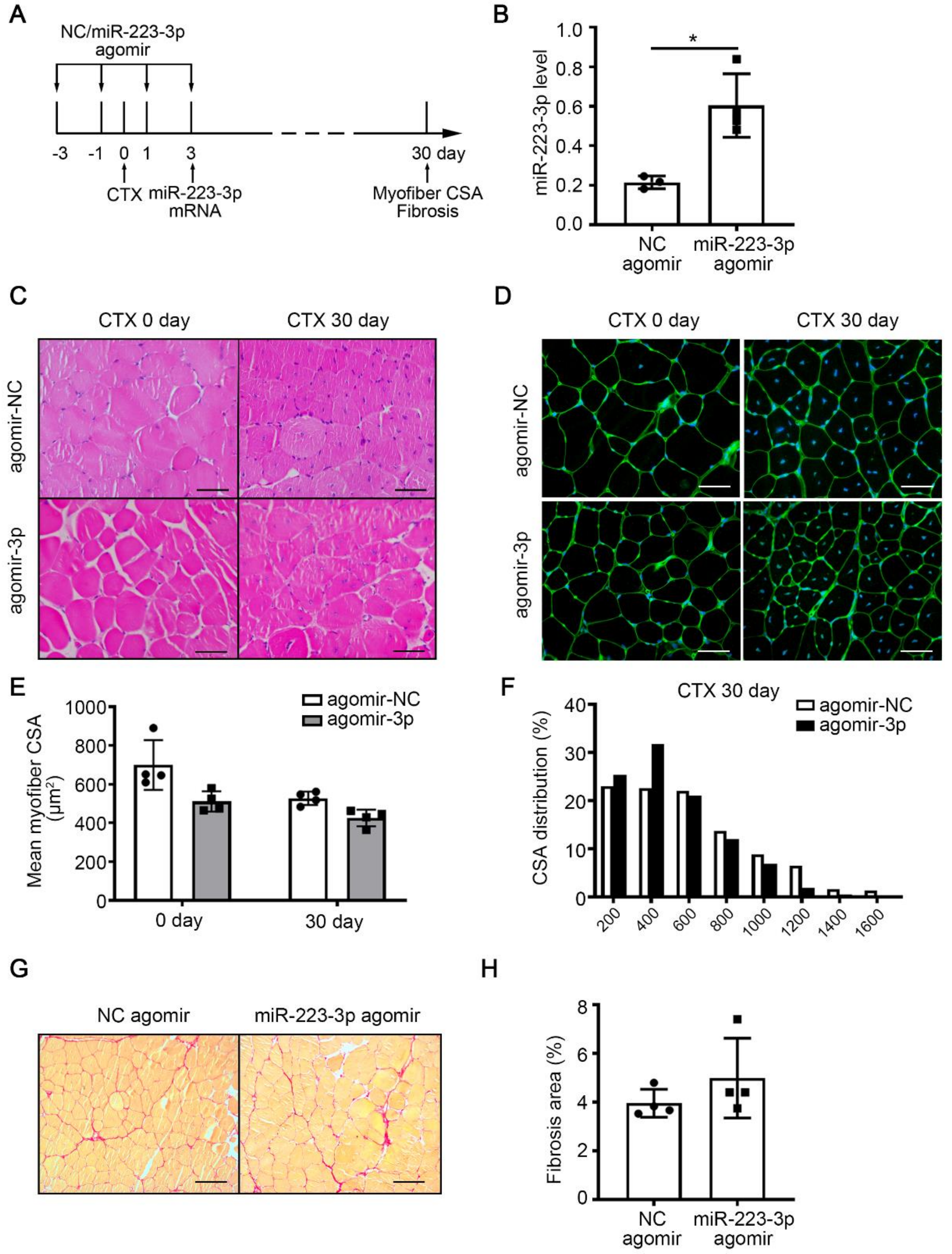
A
NC/miR-223-3p agomir
-3 -1 0 1 3
30 day
CTX miR-223-3p mRNA
Myofiber CSA Fibrosis
B
miR-223-3p level
*
NC agomir
miR-223-3p agomir
C
CTX 0 day
CTX 30 day
agomir-NC
agomir-3p
D
CTX 0 day
CTX 30 day
agomir-NC
agomir-3p
E
Mean myofiber CSA (μm²)
agomir-NC
agomir-3p
0 day
30 day
F
CTX 30 day
CSA distribution (%)
agomir-NC
agomir-3p
G
NC agomir
miR-223-3p agomir
H
Fibrosis area (%)
NC agomir
miR-223-3p agomir

Figure S2. Overexpression of miR-223-3p in wildtype mice does not affect muscle regeneration after injury

A. Schematic of the experimental design. Negative control (NC) or miR-223-3p agomir (0.6μMol·Kg^{-1}) was intravenous injected to WT muscle at 3 days and 1 day before and after CTX injury. At 3 after CTX injury, TA muscle was harvested to examine the overexpression efficiency of miR-223-3p agomir, and at 0 day and 30 days after injury, TA muscle was harvested to examine the regeneration.

B. RT-PCR assessment of miR-223-3p expression in the muscles of negative control (NC) and miR-223-3p agomir treated WT mice 3 days after CTX injury (n=3-4 per group).

C. Representative HE staining of muscles of negative control (NC) and miR-223-3p agomir treated WT mice at 0 day and 30 days after CTX injury. Scale bar, 50 μm.

D. Representative WGA staining (green) of muscles of negative control (NC) and miR-223-3p agomir treated WT mice at 0 day and 30 days after CTX injury. The nuclei were counterstained with DAPI (blue). Scale bar, 50 μm.

E. Mean myofiber cross section area (CSA) of muscles of negative control (NC) and miR-223-3p agomir treated WT mice at 0 day and 30 days after CTX injury (n=4 per group, for each sample, ≥300 myofibers were measured).

F. CSA distributions of muscles from negative control (NC) and miR-223-3p agomir treated WT mice at 30 days after CTX injury.

G. Representative Picrosirius Red staining (red) of muscles of negative control (NC) and miR-223-3p agomir treated WT mice at 30 days after CTX injury. Scale bar, 100 μm.

H. Percentages of Picrosirius Red-positive areas per field in the muscles of negative control (NC) and miR-223-3p agomir treated WT mice at 30 days after CTX injury (n=4 per group).

Data are expressed as the mean ± SD. $^{*}P < 0.05$ by unpaired two-tailed Student's t-test.

Figure S3

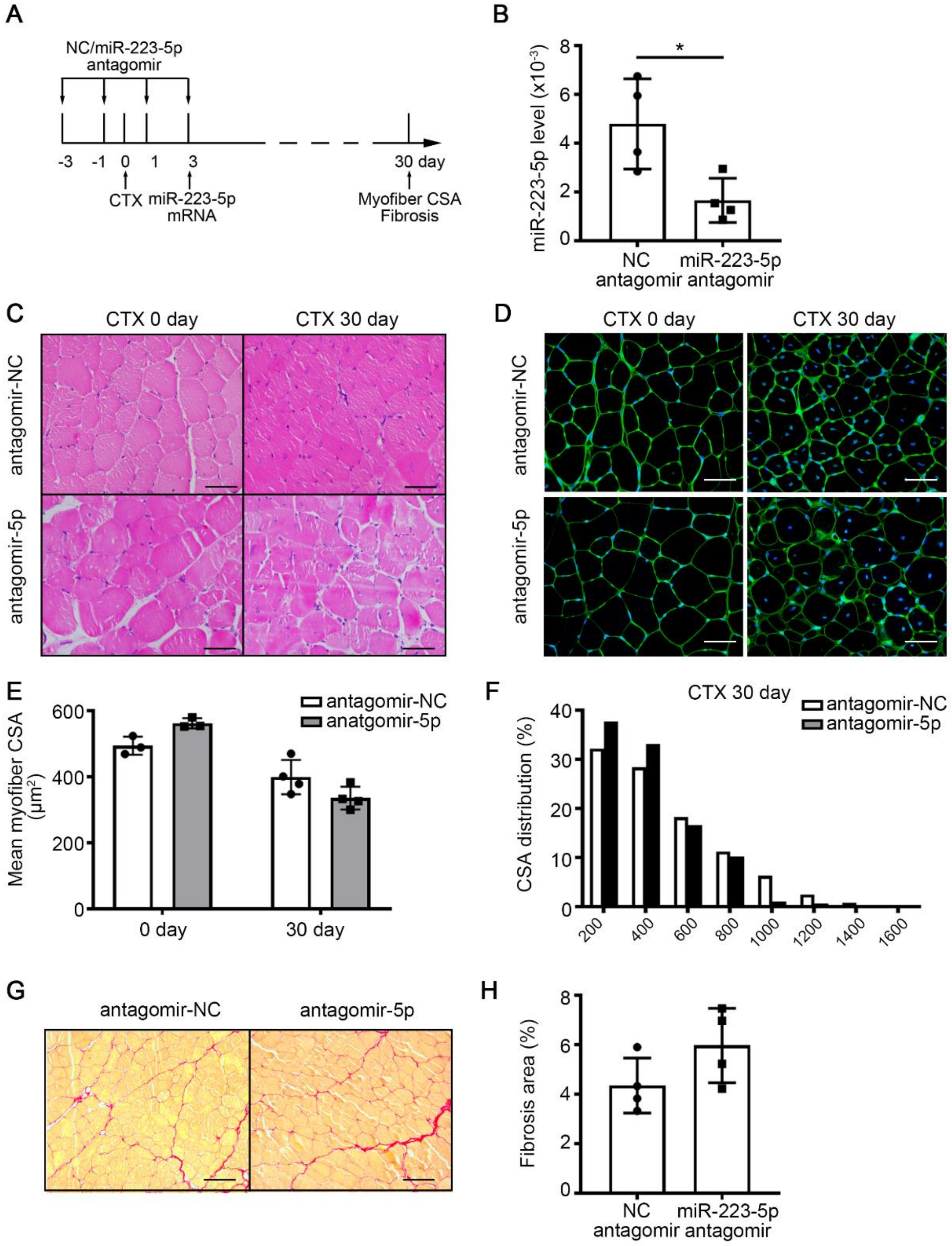
A
NC/miR-223-5p antagomir
-3 -1 0 1 3 30 day
CTX miR-223-5p mRNA
Myofiber CSA Fibrosis
B
miR-223-5p level (x10⁻³)
NC antagomir
miR-223-5p antagomir
C
CTX 0 day
CTX 30 day
antagomir-NC
antagomir-5p
D
CTX 0 day
CTX 30 day
antagomir-NC
antagomir-5p
E
Mean myofiber CSA (μm²)
antagomir-NC
anatgomir-5p
0 day
30 day
F
CTX 30 day
antagomir-NC
antagomir-5p
CSA distribution (%)
G
antagomir-NC
antagomir-5p
H
Fibrosis area (%)
NC antagomir
miR-223-5p antagomir

Figure S3. Inhibition of miR-223-5p does not affect muscle regeneration of wildtype mice

A. Schematic of the experimental design. Negative control (NC) or miR-223-5p antagomir (0.6μMol·Kg^{-1}) was intravenous injected to WT muscle at 3 days and 1 day before and after CTX injury. At 3 after CTX injury, TA muscle was harvested to examine the inhibition efficiency of miR-223-5p antagomir, and at 0 day and 30 days after injury, TA muscle was harvested to examine the regeneration.

B. RT-PCR assessment of miR-223-5p expression in the muscles of negative control (NC) and miR-223-5p antagomir treated WT mice 3 days after CTX injury (n=4 per group).

C. Representative HE staining of muscles of negative control (NC) and miR-223-5p antagomir treated WT mice at 0 day and 30 days after CTX injury. Scale bar, 50 μm.

D. Representative WGA staining (green) of muscles of negative control (NC) and miR-223-5p antagomir treated WT mice at 0 day and 30 days after CTX injury. The nuclei were counterstained with DAPI (blue). Scale bar, 50 μm.

E. Mean myofiber cross section area (CSA) of muscles of negative control (NC) and miR-223-5p antagomir treated WT mice at 0 day and 30 days after CTX injury (n=3-4 per group, for each sample, ≥300 myofibers were measured).

F. CSA distributions of muscles of negative control (NC) and miR-223-5p antagomir treated WT mice at 30 days after CTX injury.

G. Representative Picrosirius Red staining (red) of muscles of negative control (NC) and miR-223-5p antagomir treated WT mice at 30 days after CTX injury. Scale bar, 100 μm.

H. Percentages of Picrosirius Red-positive areas per field in the muscles of negative control (NC) and miR-223-5p antagomir treated WT mice at 30 days after CTX injury (n=4 per group).

Data are expressed as the mean ± SD. $^{*}P < 0.05$ by unpaired two-tailed Student's t-test.

Figure S4

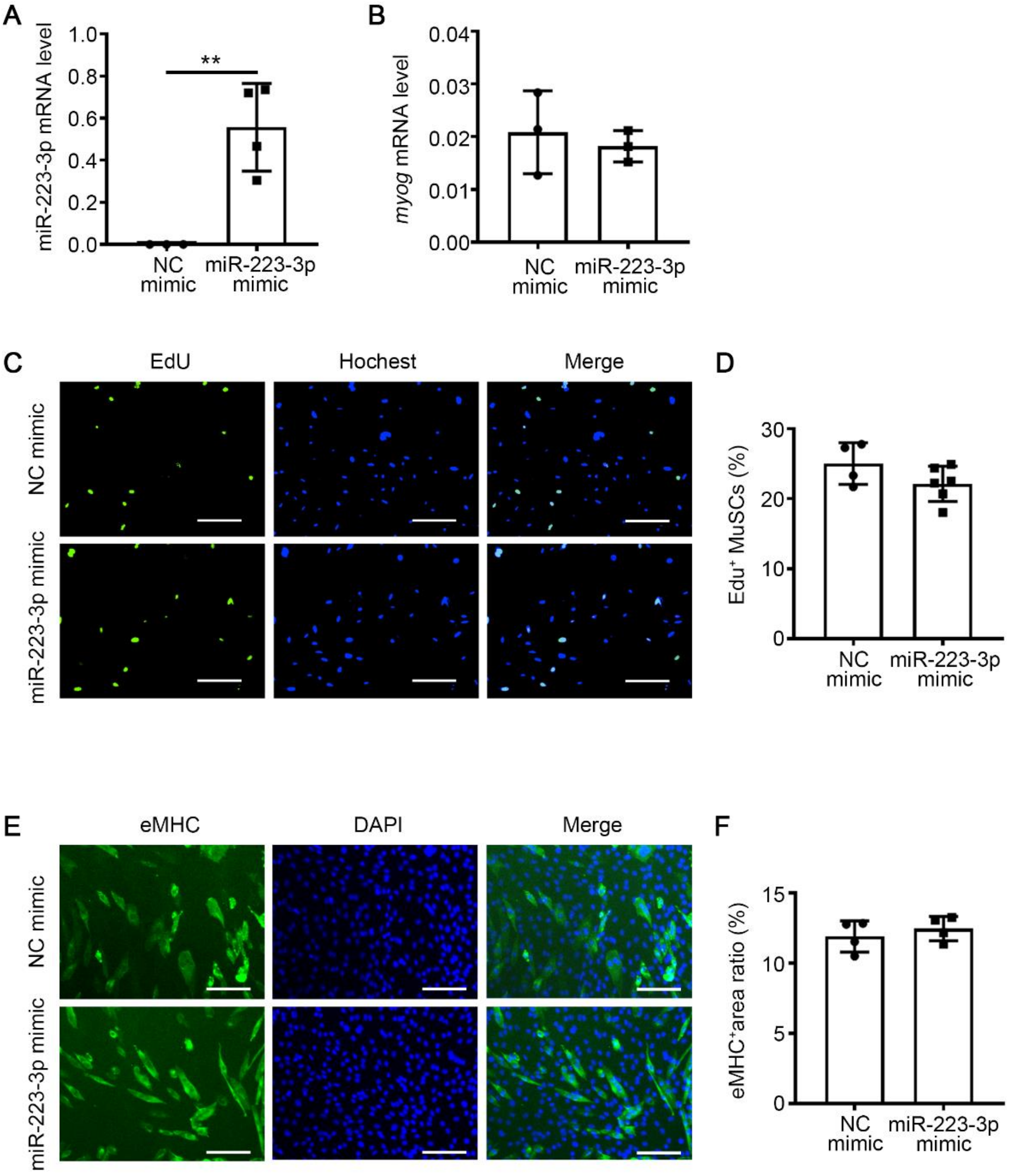
A
miR-223-3p mRNA level
1.0
0.8
0.6
0.4
0.2
0.0
**
NC mimic
miR-223-3p mimic
B
myog mRNA level
0.04
0.03
0.02
0.01
0.00
NC mimic
miR-223-3p mimic
C
EdU
Hochest
Merge
NC mimic
miR-223-3p mimic
D
Edu+ MuSCs (%)
30
20
10
0
NC mimic
miR-223-3p mimic
E
eMHC
DAPI
Merge
NC mimic
miR-223-3p mimic
F
eMHC+area ratio (%)
15
10
5
0
NC mimic
miR-223-3p mimic

Figure S4. MiR-223-3p does not affect MuSC proliferation or differentiation

A. RT-PCR assessment of miR-223-3p expression in negative control (NC) and miR-223-3p mimic transfected MuSCs.

B. RT-PCR assessment of *Myog* expression in NC or miR-223-3p mimic-transfected MuSCs after culture in differentiation medium for 3 days.

C. Representative EdU staining (green) in NC and miR-223-3p mimic-transfected MuSCs. Nuclei were counterstained with DAPI (blue). Scale bar, 100 μm.

D. Percentage of EdU-positive cells in NC and miR-223-3p mimic-transfected MuSCs (n=3-4 per group).

E. Representative eMHC immunofluorescence (green) in NC and miR-223-3p mimic-transfected MuSCs after culture in differentiation medium for 3 days. Nuclei were counterstained with DAPI (blue). Scale bar, 100 μm.

F. Percentages of eMHC-positive areas in fields of view from NC and miR-223-3p mimic-transfected MuSCs after culture in differentiation medium for 3 days (n=3-4 per group).

Data are expressed as the mean ± SD. $^{***}P < 0.001$ by unpaired two-tailed Student's t-test.

Supplemental Table1.

Table S1. Top30 upregulated miRNAs at 1 day and 3 days after injury during muscle regeneration (FC ≥ 2 and FDR ≤ 0.001)

Symbol	FPKM			Log2 FC		P-value		FDR	
	0D	1D	3D	1D-vs-0D	3D-vs-0D	1D-vs-0D	3D-vs-0D	1D-vs-0D	3D-vs-0D
mmu-miR-223-3p	822	38849	16718	5.562595	4.34612	0	0	0	0
mmu-miR-532-5p	28	20653	241	9.526709	3.105534	0	4.29E-38	0	2.22E-37
mmu-miR-142a-3p	213	10175	7620	5.578032	5.160866	0	0	0	0
mmu-miR-142a-5p	180	5493	5227	4.931525	4.859914	0	0	0	0
mmu-miR-21a-3p	28	2126	970	6.246571	5.114486	0	2.4E-221	0	2.3E-220
mmu-miR-200c-3p	38	1002	697	4.720739	4.197087	0	5.5E-141	0	4.2E-140
mmu-miR-339-5p	38	741	295	4.285402	2.956644	3.7E-218	1.43E-43	3.9E-217	7.87E-43
mmu-miR-223-5p	12	702	647	5.870365	5.752659	1.5E-237	5.6E-160	1.6E-236	4.4E-159
mmu-miR-297c-5p	0	383	435	15.22506	15.40873	4E-141	3.7E-120	3.5E-140	2.7E-119
mmu-miR-669l-5p	9	255	445	4.824428	5.627737	3.18E-80	1.1E-106	2.33E-79	7.4E-106
mmu-miR-130b-5p	11	248	430	4.494765	5.288761	3.69E-76	1.1E-100	2.67E-75	7.4E-100
mmu-miR-18a-3p	0	133	226	13.69914	14.46404	2.94E-50	4.67E-62	1.89E-49	2.85E-61
mmu-miR-466c-5p	4	82	120	4.357552	4.906891	1.88E-25	4.03E-28	9.26E-25	1.92E-27
mmu-miR-130b-3p	1	64	41	6	5.357552	1.16E-22	8.88E-11	5.36E-22	2.99E-10
mmu-miR-467a-3p	2	64	24	5	3.584963	2.24E-21	0.000027	1.02E-20	7.51E-05
mmu-miR-142b	0	60	51	12.55075	12.31628	8.91E-23	1.52E-14	4.15E-22	5.57E-14
mmu-miR-466c-3p	0	53	86	12.37178	13.07012	3.27E-20	4.79E-24	1.46E-19	2.1E-23
mmu-miR-466p-3p	0	53	86	12.37178	13.07012	3.27E-20	4.79E-24	1.46E-19	2.11E-23
mmu-miR-466b-3p	0	53	86	12.37178	13.07012	3.27E-20	4.79E-24	1.47E-19	2.11E-23
mmu-miR-141-3p	2	47	50	4.554589	4.643856	2.12E-15	8.86E-12	8.74E-15	3.04E-11
mmu-miR-7649-3p	1	43	34	5.426265	5.087463	3.94E-15	1.09E-08	1.59E-14	3.37E-08
mmu-miR-466f	1	34	44	4.523562	3.459432	4.72E-08	0.007262	1.57E-07	0.016817
mmu-miR-330-5p	1	32	37	5	5.209453	3.21E-11	1.8E-09	1.2E-10	5.81E-09
mmu-miR-147-3p	0	31	19	11.59805	10.89178	3.77E-12	7.43E-06	1.44E-11	2.13E-05
mmu-miR-466a-3p	0	27	33	11.39874	11.68825	1.1E-10	1.17E-09	4.05E-10	3.84E-09
mmu-miR-466e-3p	0	27	33	11.39874	11.68825	1.1E-10	1.17E-09	4.07E-10	3.83E-09
mmu-miR-500-5p	0	19	53	10.89178	12.37178	9.39E-08	2.33E-15	3.09E-07	8.59E-15
mmu-miR-8091	0	15	23	10.55075	11.16742	2.74E-06	6.09E-07	8.38E-06	1.81E-06
mmu-miR-301b-3p	0	14	15	10.45121	10.55075	6.38E-06	9.06E-05	1.91E-05	0.000246
mmu-miR-7062-5p	0	13	29	10.3443	11.50184	1.48E-05	7.66E-09	4.37E-05	2.4E-08

Supplemental Table2.

Gene		primer
Myod1	F	5'-AGCATAGTGGAGCGCATCTC-3'
	R	5'-GGTCTGGGTTCCCTGTTCTG-3'
Myog	F	5'-CAGCCCAGCGAGGGAATTTA-3'
	R	5'-AGAAGCTCCTGAGTTTGCCC-3'
Col1a1	F	5'-CTCTGTCACAGTCAGAGGTGT-3'
	R	5'-TTCCGACTTGCGGAGGAAAG-3'
Tgfb1	F	5'-GTCACTGGAGTTGTACGGCA-3'
	R	5'-AGCCCTGTATTCCGTCTCCT-3'
Ptprc	F	5'-CCTACACCCAGTGATGGTGC-3'
	R	5'-TTGTGCTTGGAGGGTCAGTG-3'
Itgam	F	5'-CGTGGACCTTCCAGGATGAG-3'
	R	5'-CATCTCGGAGCCTGTAGTGC-3'
Il1b	F	5'-CGTGGACCTTCCAGGATGAG-3'
	R	5'-CATCTCGGAGCCTGTAGTGC-3'
Ccl2	F	5'-CGGCTGGAGCATCCACGTGTT-3'
	R	5'-GTAGCAGCAGGTGAGTGGGGC-3'
Tnf	F	5'-TAGCCCACGTCGTAGCAAAC-3'
	R	5'-ACAAGGTACAACCCATCGGC-3'
Cxcl1	F	5'-ACTCAAGAATGGTCGCGAGG-3'
	R	5'-GTGCCATCAGAGCAGTCTGT-3'
Il6	F	5'-GGTGACAACCACGGCCTTCCC-3'
	R	5'-AAGCCTCCGACTTGTGAAGTGGT-3'
Actb	F	5'-CCTTCCAGCAGATGTGGATCA-3'
	R	5'-CTCAGTAACAGTCCGCCTAGAA-3'
